# Impact of invasive mechanical ventilation support on renal function in critically ill patients

**DOI:** 10.1590/1980-220X-REEUSP-2025-0309en

**Published:** 2026-02-27

**Authors:** João Marcos Santos Rocha, Tayse Tâmara da Paixão Duarte, Michelle Zampieri Ipolito, Walterlânia Silva Santos, Paulo Percio Mota Magro, Marcia Cristina da Silva Magro

**Affiliations:** 1 Universidade de Brasília, Faculdade de Ciências e Tecnologias em Saúde, Brasília, DF, Brazil.; 2 Instituto Federal de Brasília, Brasília, DF, Brazil.

**Keywords:** Acute Kidney Injury, Respiration, Artificial, Intensive Care Units, Organ Dysfunction Scores

## Abstract

**Objective:**

To assess the impact of invasive mechanical ventilation on renal function and to verify the predisposing factors for the development of acute kidney injury in relation to the use of invasive mechanical ventilation in intensive care.

**Method:**

An observational, retrospective, quantitative cohort study. The sample was non-probabilistic, of convenience, and consisted of 51 patients. Patient severity was assessed using the Simplified Acute Physiology Score and Sequential Organ Failure Assessment. All tests were two-tailed, and p < 0.05 was considered statistically significant.

**Results:**

Among patients with kidney injury on invasive mechanical ventilation, 41.2% presented with severe kidney injury (stage 3 of the Kidney Disease Initiative Global Outcomes). The duration of mechanical ventilation was longer in patients with acute kidney injury compared to those without renal impairment (19 versus four days).

**Conclusion:**

The impact of mechanical ventilation in critically ill patients was evidenced by the higher prevalence of severe acute kidney injury. Invasive ventilatory support was more prevalent among older adults, highlighting the severity of the patients based on the Simplified Acute Physiology Score and Sequential Organ Failure Assessment scores, and consequently a higher risk of death.

## INTRODUCTION

Acute kidney injury (AKI) is a complex clinical syndrome that significantly influences the disease process, worsening the outcome for a large number of hospitalized patients, increasing morbidity and mortality, and prolonging hospital stay^(1)^.

In low- and middle-income countries, infections and hypovolemic shock are the predominant causes of AKI^(2)^. In high-income countries, AKI occurs primarily in older hospitalized patients and is related to sepsis, medications, or invasive procedures^(2)^. From this perspective, severe AKI has proven prevalent in Intensive Care Units (ICUs), with a higher incidence over the last few decades in older patients, which contributes to high mortality and results in high costs related to the management of difficult-to-prevent AKI^(3)^.

The Kidney Disease Improving Global Outcome (KDIGO) clinical practice guidelines describe AKI as an increase in serum creatinine (SCr) ≥ 0.3 mg/dL over two days, or an increase in SCr ≥ 1.5 times the baseline value, or a urine output < 0.5 mL/kg/h over six hours^(4)^. The incidence rate of AKI in hospitalized patients varies between 7%^(5)^ and 23%^(5)^. However, in intensive care settings, the occurrence rises to 39%, with mortality around 49%^(6)^.

Observing the development of AKI in intensive care settings, we found greater patient severity, prolonged hospital stay, increased use of antibiotics and vasoactive drugs, and a need for invasive mechanical ventilation (IMV)^(7)^.

In this context, several prognostic scores have been developed and used in intensive care, encompassing a variety of clinical characteristics and laboratory data, with the aim of assessing patient severity and guiding measures and interventions. The Simplified Acute Physiology Score 3 (SAPS 3), Sequential Organ Failure Assessment (SOFA), and Nursing Activities Score (NAS) have been used for prognostic assessment of severity. They incorporate different clinical, laboratory, and physiological variables to provide an estimate of disease severity, mortality risk, and nursing workload for critically ill patients^(8)^. IMV is a type of ventilatory support frequently used in the ICU, in which there is total or partial replacement of spontaneous ventilation. In Brazil, approximately 36.2% of critically ill patients are on mechanical ventilation for various reasons^(9)^. Mortality in patients on mechanical ventilation tends to increase as multiple organ failure occurs, including kidney failure^(8)^. This scenario highlights the significant difficulties in the clinical management of these patients, reinforcing the importance of an accurate prognostic assessment tailored to each patient’s specific needs^(10)^. However, the magnitude of this occurrence in critically ill and older patients is still poorly described, as is the impact of mechanical ventilation on renal function, which reinforces the importance of improving knowledge about ventilatory factors that can impact suboptimal renal function^(11)^.

Although the association between IMV and the occurrence of AKI in critically ill patients is recognized, the mechanisms underlying this interaction are not yet fully elucidated, especially regarding the influence of different ventilatory parameters on renal perfusion. Therefore, the present study aims to assess the impact of IMV on renal function and to verify the predisposing factors for the development of AKI in relation to the use of IMV in intensive care.

## METHOD

### Study Design, Period and Location

This is an observational cohort study^(12)^, retrospective with a quantitative approach^(12)^, referring to the period from January to December 2023, based on STrengthening the Reporting of OBservational studies in Epidemiology guidelines^(13)^. It was developed in a mixed ICU (clinical and coronary), consisting of 19 surgical and clinical beds in a tertiary teaching hospital located in midwestern Brazil.

### Sample and Inclusion Criteria

**Figure 1:**
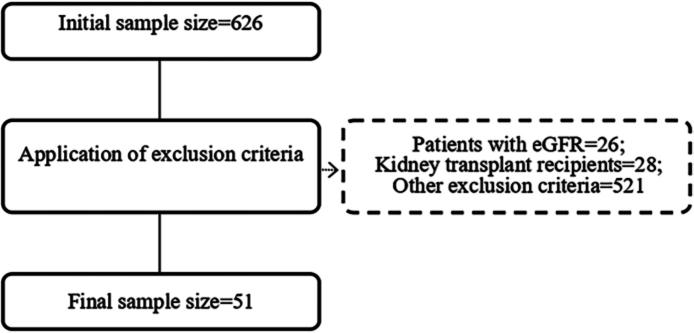
Flowchart regarding participant selection criteria.

The population consisted of medical and surgical patients admitted to the ICU. The sample was non-probabilistic, based on convenience, and comprised 51 patients, as detailed in the flowchart (Figure 1).

Patients aged ≥ 18 years with sustained changes in SCr ≥ 0.3 mg/dL from baseline for at least 48 hours, as per the KDIGO classification^(2,4)^, were included, as well as inpatients in the general or coronary care departments who underwent IMV for at least 48 hours.

Patients who were hospitalized more than once, with an estimated glomerular filtration rate (eGFR), according to the Chronic Kidney Disease Epidemiology Collaboration (CKD-Epi), less than 30 mL/min, without SCr measurement, with a length of stay in the ICU of less than 48 hours, and those in palliative care and/or kidney transplant recipients, and patients on a chronic dialysis program were excluded.

### Data Collection Procedure

Data were collected using an instrument developed in the form of a structured questionnaire with closed-ended items, subdivided into five domains consisting of the following variables: identification (weight, ethnicity, height, age, sex); outcome; past history (comorbidities); current history (renal function, laboratory data, IMV, ventilatory mode, positive end-expiratory pressure (PEEP), inspired oxygen fraction, tidal volume, respiratory rate, vasoactive drugs/antibiotics in the ICU); and scores (NAS, SAPS 3, and SOFA).

Creatinine levels were measured using the kinetic reaction method with Jaffé buffer without deproteinization, and SCr samples were collected by professionals according to the unit’s routine.

Consultation and extraction of patient medical records, for recording study variables in the questionnaire, and subsequent storage in a database created in Microsoft Office Professional Plus 2019 were performed by pairs, two independent researchers, aiming to mitigate the occurrence of biases and increase the reliability of results.

Daily monitoring of laboratory and clinical data of patients on IMV for 15 consecutive days, and subsequently at the end of their ICU stay, verified the occurrence of AKI and other clinical developments. An older adult was defined as an individual aged ≥ 60 years^(14)^.

Several concepts were adopted for standardizing data collection. The prognostic scores used followed several data sources, such as SAPS 3, defined as an instrument composed of parameters tangent to an acute and prior physiological score capable of generating a prognosis for severity and risk of death in patients under care in ICUs^(15)^. The SOFA score is used to estimate mortality risk based on the continuous assessment of organ dysfunction at physiological and laboratory levels^(16)^.

To assess the nursing team’s workload, NAS was used as an instrument capable of assessing nursing professionals’ workload in an intensive care setting, proposing interpretations regarding team sizing and the maintenance of specific care^(17)^.

IMV was defined as ventilatory support which, through an endotracheal tube or tracheostomy cannula, provides total or partial replacement of spontaneous ventilation^(18)^, with prolonged mechanical ventilation considered as ventilatory support for a period exceeding seven days^(19)^.

Cases requiring IMV were identified based on the oxygen device recorded as “tracheostomy, mechanical ventilation” or “endotracheal tube” in patients’ medical record at any point during their ICU stay. IMV duration was calculated as the sum of the days during which a patient was recorded as requiring IMV, as defined above.

Renal function assessment was based on SCr criteria established in KDIGO guidelines, due to the absence of a systematic and accurate record of urine volume, which classifies the severity of AKI into the following stages: KDIGO 1 – SCr value greater than or equal to 0.3 mg/dL; KDIGO 2 – 2.0 to 2.9-fold increase in SCr level compared to baseline; KDIGO 3 – 3-fold increase in SCr level compared to baseline, creatinine value ≥ 4 mg/dL, or initiation of renal replacement therapy^(5)^.

The baseline SCr level was the lowest value in the most recent 365-day period prior to hospitalization for AKI, or the lowest value in the first seven days prior to hospitalization^(20)^. eGFR values were calculated by CKD-Epi^(4)^.

Renal function recovery was calculated in patients with AKI by the ratio of SCr levels at the time of assessment to baseline SCr levels. There are different levels of recovery. In this study, full renal function recovery was assessed: when SCr returns to the baseline creatinine level^(20)^.

Acute kidney disease (AKD) was identified when persistent AKI was observed for a period of seven to 90 days after exposure to an aggressive event^(20)^.

### Statistical Analysis

Before data analysis, the normality of continuous data was tested using the Kolmogorov-Smirnov test. An asymmetrical sample was found. Therefore, data were expressed as median (interquartile range), and the difference among groups was compared using the Mann-Whitney U test for independent samples. Categorical variables were expressed as numbers (percentages) and compared using the chi-square test or Fisher’s exact test.

Statistical analysis was conducted using the Statistical Package for the Social Sciences 23.0. All tests were two-tailed, and p < 0.05 was considered statistically significant.

### Ethical Aspects

The study was conducted in accordance with national and international ethical guidelines. It was approved by the Research Ethics Committee of the Faculty of Health Sciences and Technologies, *Universidade de Brasília*, under Certificate of Presentation for Ethical Consideration 61486022.0.0000.8093 and Opinion 7,165,243. All included patients signed the Informed Consent Form.

## RESULTS

Fifty-one brown male patients, undergoing IMV, were followed. The median age was 61 years, predominant in both the groups with and without AKI. Overweight was a characteristic of patients without AKI [27.8 (23.2–32.7)] kg/m^2^. Hypertension (45.1%) was the most frequent comorbidity among patients. Patients hospitalized for clinical treatment predominated (82.4%). Carbapenem antibiotics (52.9%) were commonly used, and norepinephrine (80.4%) was the most frequently administered vasoactive drug (Table 1). It is noteworthy that 28.9% of patients with AKI required renal support therapy. More than half of the patients on mechanical ventilation required PEEP between 5 and 10 cm/H_2_O (90.2%). It is also noteworthy that 50% of patients with AKI remained on IMV for more than seven days. In the group with AKI, death affected more than 50% of patients. The median overall length of hospital stay was 15 days (Table 1).

**Table 1:** Demographic and clinical characterization – Federal District, Brasília, Brazil, 2023.

Variables	Without AKI (n = 13)	With AKI (n = 38)	Total (n = 51)
Male sex *n (%)*	9 (69.2)	22 (57.9)	31 (60.8)
Age (years) *mean ± SD; median (IQR)*	58 ± 17 63 (43–69)	55 ± 18 61 (43–69)	56 ± 18 61 (44–69)
BMI (kg/m^2^) *mean ± SD; median (IQR)*	27.6 ± 5.0 27.8 (23.2–32.7)	23.7 ± 6.2 23.4 (18.3–28.7)	24.9 ± 6.0 24.6 (20.8–29.6)
Race *n (%)*	
White	1 (7.7)	5 (13.2)	6 (11.8)
Black	0 (0.0)	1 (2.6)	1 (2.0)
Brown	10 (76.9)	27 (71.1)	37 (72.5)
Other	1 (7.7)	0 (0.0)	1 (2.0)
Not declared	1 (7.7)	5 (13.2)	6 (11.8)
Comorbidities *n (%)*	
Diabetes mellitus	4 (30.8)	8 (21.1)	12 (23.5)
Hypertension	6 (46.2)	17 (44.7)	23 (45.1)
COPD	2 (15.4)	6 (15.8)	8 (15.7)
AMI	2 (15.4)	8 (21.1)	10 (19.6)
ARF	1 (7.7)	8 (21.1)	9 (17.6)
Smoking	4 (30.8)	14 (36.8)	18 (35.3)
Alcoholism	4 (30.8)	7 (18.4)	11 (21.6)
Reason for hospitalization *n (%)*	
Clinical	11 (84.6)	31 (81.6)	42 (82.4)
Surgical	2 (15.4)	7 (18.4)	9 (17.6)
Medications *n (%)*	
Norepinephrine	8 (61.5)	33 (86.8)	41 (80.4)
Vasopressin	4 (30.8)	17 (44.7)	21 (41.2)
Amikacin (aminoglycoside)	2 (15.4)	8 (21.1)	10 (19.6)
Vancomycin (glycopeptide)	3 (23.1)	17 (44.7)	20 (39.2)
Meropenem (carbapenem)	6 (46.2)	21 (55.3)	27 (52.9)
Tazocin (lactamase)	4 (30.8)	13 (34.2)	17 (33.3)
RRT *n (%)*	0 (0.0)	11 (28.9)	11 (21.6)
PEEP *n (%)*	
Group 1 (highest PEEP <= 5)	0 (0.0)	3 (7.9)	3 (5.9)
Group 2 (5 < highest PEEP <= 10)	13 (100.0)	33 (86.8)	46 (90.2)
Group 3 (highest PEEP > 10)	0 (0.0)	2 (5.3)	2 (3.9)
ICU outcome *n (%)*	
Death	5 (38.5)	22 (57.9)	27 (52.9)
Clinical discharge	0 (0.0)	3 (7.9)	3 (5.9)
Discharge home	1 (7.7)	1 (2.6)	2 (3.9)
Referred to another hospital unit	7 (53.8)	12 (31.6)	19 (37.3)
Ventilation mode *n (%)*	
Assisted and controlled	15 (115.4)	44 (115.8)	59 (115.7)
Pressure support	10 (76.9)	27 (71.1)	37 (72.5)
IMV time (>seven days) *n (%)*	4 (30.8)	19 (50.0)	23 (45.1)
Length of stay *mean ± SD; median* (IQR)	22 ± 16 18 (8–34)	19 ± 14 15 (8–29)	20 ± 14 15 (8–30)

Legend: SD – standard deviation; IQR – interquartile range; BMI – Body Mass Index; COPD – chronic obstructive pulmonary disease; AMI – acute myocardial infarction; ARF – acute respiratory failure; RRT – renal supportive therapy; PEEP – positive end-expiratory pressure; IMV – invasive mechanical ventilation; ICU – Intensive Care Unit; AKI – acute kidney injury.

Patients with AKI showed significant changes in serum lactate levels (p-value = 0.04), with significantly elevated SOFA and NAS scores observed in the AKI group on IMV (p-value = 0.003 versus 0.02). Hemoglobin levels were generally reduced (p-value = 0.02). SAPS 3 and SOFA scores remained elevated during ICU stay, representing a high risk of mortality (Table 2).

**Table 2:** Characterization of clinical and laboratory severity – Federal District, Brasília, Brazil, 2023.

Variables	Without AKI (n = 13)	With AKI (n = 38)	Total (n = 51)	p-value
Mean lactate (mg/dL) *mean ± SD; median (IQR)*	23.9 ± 27.5 13 (11–15)	26.5 ± 24.1 16 (13–24)	25.9 ± 24.7 15 (13–24)	0.04
Mean Hb (%) *mean ± SD; median (IQR)*	4.8 ± 2.2 4.7 (3.2–6.3)	6.7 ± 2.5 6.4 (5.3–8.2)	6.2 ± 2.5 6.1 (4.7–8.1)	0.02
PEEP (cm/H_2_O) *maximum mean ± SD; median (IQR)*	8.4 ± 1.5 8 (8–10)	8.0 ± 1.8 8 (6–10)	8.1 ± 1.8 8 (7–10)	0.4
SAPS 3 *mean ± SD; median (IQR)*	69 ± 15 74 (58–83)	68 ± 13 69 (62–75)	68 ± 13 69 (59–75)	0.6
Maximum NAS *mean ± SD; median (IQR)*	105.7 ± 6.4 107.3 (104.5–107.3)	113.3 ± 11.1 111.2 (107.3–115.0)	112 ± 11 108.4 (107.3–115.0)	0.02
Maximum SOFA *mean ± SD; median (IQR)*	6.3 ± 2.3 6.0 (4.5–7.5)	9.0 ± 2.6 9.0 (7.5–11.0)	8.3 ± 2.8 8 (6–10)	0.003

Legend: SD – standard deviation; IQR – interquartile range; Hb – hemoglobin; PEEP – positive end-expiratory pressure; SAPS 3 – Simplified Acute Physiology Score 3; NAS – Nursing Activities Score; SOFA – Sequential Organ Failure Assessment; AKI – acute kidney injury.

The results show that most patients (41.2%) developed more severe AKI (KDIGO 3), while 33.3% of patients developed mild to moderate renal impairment (KDIGO 1 and KDIGO 2) (Table 3).

**Table 3:** Distribution of patients according to the severity of renal impairment – Federal District, Brasília, Brazil, 2023.

KDIGO	Creatinine criterion n (%)
No kidney problems	13 (25.5%)
KDIGO 1	8 (15.7%)
KDIGO 2	9 (17.6%)
KDIGO 3	21 (41.2%)

Legend: KDIGO – Kidney Disease Improving Global Outcome.

It is noteworthy that seven (18.8%) had AKD characterized by persistent AKI for more than seven days. Of the total of 38 patients with AKI, 21 (55.3%) patients recovered renal function by the 15^th^ day of follow-up.

Table 4 shows that IMV duration did not significantly influence the occurrence of deaths and acute respiratory infections. The results show that there was no significant difference between SOFA, SAPS 3, and NAS scores, regardless of IMV duration. Maximum PEEP was significantly higher in the group that remained on prolonged IMV (greater than seven days) (p-value = 0.04) and had a longer ICU stay (p-value = 0.005).

**Table 4:** Association between invasive mechanical ventilation time and outcome variables – Federal District, Brasília, Brazil, 2023.

Variables	IMV time <= seven days (n = 28)	IMV time > seven days (n = 23)	p-value
Deaths *n (%)*	14 (50.0)	13 (56.5)	0.6
Mean lactate (mg/dL)	15 (53.6)	3 (13.0)	0.002
pH *(median (IQR))*	7.39 (7.31–7.43)	7.42 (7.37–7.45)	0.13
AKI *n (%)*	19 (67.9)	19 (82.6)	0.2
AKI KDIGO 2 or KDIGO 3 *n (%)*	14 (50.0)	16 (69.6)	0.2
Maximum PEEP *mean ± SD; median (IQR)*	7.7 ± 1.8 8 (6–9)	8.6 ± 1.5 8 (8–10)	0.04
SAPS3 *mean ± SD; median (IQR)*	69 ± 16 69 (55–79)	68 ± 9 69 (64–74)	0.8
Maximum NAS *mean ± SD; median (IQR)*	110.9 ± 10.7 107.6 (106.7–115.0)	112.8 ± 11.0 109.1 (107.3–115.0)	0.4
Maximum SOFA *mean ± SD; median (IQR)*	7.8 ± 2.9 8 (5–10)	9.0 ± 2.6 9 (7–10)	0.2
Length of stay *mean ± SD; median (IQR)*	16 ± 14 10 (7–24)	24 ± 14 20 (15–34)	0.005

Legend: SD – standard deviation; IQR – interquartile range; IMV – invasive mechanical ventilation; AKI – acute kidney injury; PEEP – positive end-expiratory pressure; SAPS 3 – Simplified Acute Physiology Score 3; KDIGO – Kidney Disease Improving Global Outcome; NAS – Nursing Activities Score; SOFA – Sequential Organ Failure Assessment.

## DISCUSSION

A higher prevalence of IMV was observed in critically ill male patients with AKI, especially in the most severe cases, classified as KDIGO 3. Invasive ventilatory support was more frequent among older adults, reflecting greater clinical severity, as evidenced by SAPS 3 and SOFA scores, and indicating a higher risk of mortality. Furthermore, ventilation time exceeding seven days was associated with prolonged hospital stays and the need for higher PEEP levels.

SOFA and SAPS 3 scores are widely used to assess severity and mortality risk in critically ill patients, and have demonstrated acceptable prognostic ability to predict the development of AKI^(21,22)^. This association stems from the fact that higher scores reflect greater organ dysfunction, an exacerbated inflammatory response, oxidative stress, and reduced renal perfusion^(23)^. In parallel, NAS also showed elevated levels, indicating a greater workload for the nursing staff, especially in patients with AKI. This finding, observed in our study, may be related to the need for vasoactive drugs, biochemical alterations, prolonged hospital stay, and invasive ventilatory support, which require longer monitoring times and continuous intensive care^(22)^.

Despite the widespread use of IMV as a life-saving technology, the longer its duration, the greater the risk of complications and mortality. In our study, patients with longer IMV durations more frequently developed AKI. Death occurred in more than half of patients on IMV, both with and without AKI, highlighting the importance of prioritizing the initiation of care processes for weaning patients from IMV as quickly as possible^(19)^.

In line with these results, a previous study demonstrated that there is an inflammatory interaction between the lung and the kidney, such that injury to one organ contributes to injury and inflammation in the other. Therefore, patients with AKI may be more predisposed to ventilator-induced lung injury, thus being one of the main contributors to multiple organ failure and death in critically ill patients^(23)^. In our study, longer mechanical ventilation time impacted prolonged hospital stay (p = 0.005).

IMV duration and length of stay in the ICU can be considered, at least partially, indicators of quality of care. It has been reported that 5 to 20% of ICU patients require mechanical ventilation for more than seven days. Among critically ill patients, the mean length of stay in the ICU ranges from 2 to 13 days, depending on the patient’s profile and the severity of the case. In this study, the median length of stay for patients on IMV with and without AKI was 15 days. A multicenter study conducted in Brazil showed that patients on IMV had an average length of stay in the ICU of ten days, with high hospital mortality (42%)^(24)^. In our study, death from any cause affected more than 50% of patients on IMV.

Despite the negative effects of IMV on renal function, as well as other factors such as reduced hemoglobin and altered lactate levels identified in the current investigation, renal recovery was observed in more than half of 74.5% of patients affected by AKI by the 15^th^ day of follow-up. This is an expected condition after adequate treatment, although approximately one in five patients with AKI developed AKD, meaning AKI persists for more than seven days^(20)^. One contributing factor may have been the significant elevation of lactate. In the context of critically ill patients, such as those with AKI, elevated lactate levels often signify tissue hypoperfusion and insufficient oxygenation, predisposing to renal dysfunction^(10)^. Furthermore, anemia can also prove to be a factor that increases the chance of mortality, a condition identified among the patients in our study. It should be noted that anemia can also result in incompatibility between oxygen supply and consumption, particularly in organs that depend primarily on oxidative metabolism, such as renal tubular epithelial cells, favoring the occurrence of AKI in critically ill patients^(25)^.

Current studies have shown that the kidney is an important gluconeogenic organ, in which lactate is the primary substrate for renal gluconeogenesis under physiological conditions. During AKI, alterations in glycolysis and gluconeogenesis in the kidneys significantly disrupt the metabolic balance of lactate, which impacts AKI severity and prognosis, and may even contribute to higher mortality in this group compared to the group without AKI^(24,26)^.

In this regard, the use of clinical prediction models to detect the onset of severe AKI in the first week of ICU admission during the start of IMV may be a promising alternative for early detection, given the frequent occurrence of AKI in predominantly older patients and the worsening prognosis of patients expressed by changes in SAPS 3 and SOFA^(22)^, as verified in our investigation.

In the present study, most patients required noradrenaline and vasopressin, potent vasoconstrictors, and almost 90% of patients with AKI required PEEP greater than five and less than or equal to 10 cmH_2_O. Higher PEEP was found more frequently in patients with a longer need for IMV, a condition also evidenced in a retrospective cohort of patients admitted to the ICU with COVID-19^(27)^.

In general, mechanical ventilation can cause significant hemodynamic disturbances, especially due to PEEP’s action, which increases intrathoracic pressure, reduces venous return, and consequently, cardiac output. Studies, such as that of Ottolina *et al*., have demonstrated that the use of high PEEP can increase the risk of developing AKI by up to five times. Although the association between ventilation and AKI is well documented, the exact mechanism is not yet fully understood. Among the most accepted hypotheses are reduced glomerular perfusion, decreased renal blood flow, and the activation of systemic inflammatory mediators, which together contribute to kidney injury^(27)^.

It is also worth noting that the use of higher doses of vasopressors may be associated with both an increased inflammatory response and the hemodynamic effects of positive pressure ventilation at higher PEEP levels. Elevated PEEP values can compromise renal perfusion and eGFR more markedly, favoring the development of renal dysfunction. In our analysis, most patients used a maximum PEEP of 10 cmH_2_O, and only a smaller proportion required higher values. Experimental studies highlight that, although the adverse effects of PEEP are more evident in the respiratory system, significant extrapulmonary repercussions can also occur, including impairments to renal function^(27,28)^. Once an AKI has developed, the collaborative action of the interprofessional team, especially nursing staff, should focus on preventing new injuries, avoiding the use of nephrotoxic medications, and ensuring the appropriate dosage of pharmacological therapies^(29)^.

In this vein, meta-analysis highlights that early recognition of AKI can favor its reversal or even the renal function recovery, contributing to patients receiving adequate fluids and medications, thus preventing the worsening of renal function or any possibility of toxicity. AKI care packages allow non-nephrologists to take rapid measures to prevent and treat AKI^(30)^.

Hospitalized patients with more severe conditions, as in our study, have a higher risk of developing AKI, which is associated with high mortality. Consensus guidelines for AKI recommend immediate treatment, including maintaining perfusion pressure, improving fluid status, preventing nephrotoxins, and preventing hyperglycemia^(30)^.

The applicability and effectiveness of prognostic scores in critically ill patients with AKI may vary due to the characteristics of this population, such as the presence of multiple comorbidities, hemodynamic instability, and exposure to intensive care. In the current study, we addressed SAPS 3, SOFA, and NAS, the latter referring to the nursing staff’s workload. Although they did not show a significant relationship with IMV duration, they did show a significant relationship with AKI, as in Brazilian prospective cohort studies^(22,31)^. It has been observed that the discriminatory power of NAS indicates a high demand for care from the nursing staff, and a higher NAS value has even been associated with the development of AKI in critically ill patients^(21,22)^.

Our study has limitations, such as being a single-center cohort, and generalizability of our results is limited, with the presence of a risk of measurement bias and a small sample size.

The findings of this study contribute significantly to strengthening nursing practice in ICUs by highlighting the association between IMV and the occurrence of AKI, especially in more severe stages of the KDIGO classification. This knowledge reinforces the importance of continuous clinical monitoring by the nursing staff in the face of hemodynamic changes and laboratory markers of renal function in critically ill patients on ventilatory support.

Furthermore, the results highlight the need to implement nursing protocols that integrate early monitoring of renal function with respiratory care, promoting timely interventions that can minimize AKI progression. Such practices broaden the scope of intensive care nurses’ role, favoring evidence-based clinical decisions, with a direct impact on patient safety and outcomes.

## CONCLUSION

The impact of mechanical ventilation on critically ill patients was evidenced by the higher prevalence of severe AKI (KDIGO 3) and AKD. Invasive ventilatory support was more prevalent among older adults, highlighting the severity of patients based on SAPS 3 and SOFA scores and a consequent higher risk of death. Some factors, such as biochemical alterations in lactate and hemoglobin, proved significant among patients on IMV with AKI.

Mechanical ventilation duration did not necessarily result in a greater workload for the nursing staff, given that the care of patients with AKI, in itself, impacted the increase in NAS, regardless of the length of time they remained on mechanical ventilation. It is important to highlight that continuous monitoring by the nursing staff is fundamental for the early detection of hemodynamic changes and laboratory markers of renal function in more severely ill patients under ventilatory support. The development and implementation of protocols are essential, allowing for appropriate interventions that can minimize AKI progression, improve clinical outcomes, and increase patient safety.

## Data Availability

The entire dataset supporting the results of this study was published in the article itself.
