# Supplementary material for: Impact of invasive mechanical ventilation support on renal function in critically ill patients
Source: Rev Esc Enferm USP. 2026 Feb 27;60:e20250309. doi: 10.1590/1980-220X-REEUSP-2025-0309en (PMC12965746; doi:10.1590/1980-220X-REEUSP-2025-0309en)
Supplement: Translation

# Translation: Impacto do suporte ventilatório mecânico invasivo na função renal de pacientes críticos

**DOI:** 10.1590/1980-220X-REEUSP-2025-0309pt

**Authors:** João Marcos Santos Rocha, Tayse Tâmara da Paixão Duarte, Michelle Zampieri Ipolito, Walterlânia Silva Santos, Paulo Percio Mota Magro, Marcia Cristina da Silva Magro

**Affiliations:** 1 Universidade de Brasília, Faculdade de Ciências e Tecnologias em Saúde, Brasília, DF, Brasil.; 2 Instituto Federal de Brasília, Brasília, DF, Brasil.

**Keywords:** Injúria Renal Aguda, Respiração Artificial, Unidades de Terapia Intensiva, Escores de Disfunção Orgânica

## Abstract

**Objetivo:**

Avaliar o impacto do suporte ventilatório mecânico invasivo na função renal e verificar os fatores predisponentes para o desenvolvimento de injúria renal aguda em relação à utilização de ventilação mecânica invasiva em terapia intensiva.

**Método:**

Estudo observacional de coorte, retrospectivo e quantitativo. A amostra foi não probabilística, de conveniência e constituída de 51 pacientes. A gravidade do paciente foi avaliada pelos escores *Simplified Acute Physiology Score* e *Sequential Organ Failure Assessment*. Todos os testes foram bicaudais, e *p* < 0,05 foi considerado significância estatística.

**Resultados:**

Entre os pacientes com injúria renal em ventilação mecânica invasiva, 41,2% apresentaram injúria renal grave (estágio 3 do *Kidney Disease Improving Global Outcome*). O tempo de ventilação mecânica foi maior nos pacientes com injúria renal aguda quando comparado àqueles sem comprometimento renal (19 *versus* quatro dias).

**Conclusão:**

O impacto da ventilação mecânica em pacientes críticos foi evidenciado pela maior prevalência de injúria renal aguda grave. O suporte ventilatório invasivo foi mais prevalente entre os idosos, evidenciando a gravidade dos pacientes a partir dos escores *Simplified Acute Physiology Score* e *Sequential Organ Failure Assessment*, e consequente maior risco de morte.

## INTRODUÇÃO

A injúria renal aguda (IRA) é uma síndrome clínica complexa que influencia significativamente o processo da doença, piorando a evolução de um grande número de pacientes hospitalizados, impactando o aumento da morbimortalidade e prolongando o tempo de permanência hospitalar^(1)^.

Nos países de baixa e média renda, as infecções e o choque hipovolêmico são as causas predominantes de IRA^(2)^. Nos países de alta renda, a IRA ocorre principalmente em pacientes idosos hospitalizados, e está relacionada à sepse, medicamentos ou procedimentos invasivos^(2)^. Nessa perspectiva, a IRA grave tem se mostrado prevalente em Unidades de Terapia Intensiva (UTIs), com maior incidência ao longo das últimas décadas em pacientes idosos, a qual corrobora para uma alta mortalidade, resultando em custos elevados relacionados ao manejo da IRA de difícil prevenção^(3)^.

Diretrizes da prática clínica do *Kidney Disease Improving Global Outcome* (KDIGO) descrevem a IRA como aumento da creatinina sérica (CrS) ≥ 0,3 mg/dl em dois dias ou elevação da CrS ≥ 1,5 vezes em relação ao valor basal ou ainda débito urinário < 0,5 ml/kg/h em seis horas^(4)^. A taxa de incidência de IRA em pacientes hospitalizados varia entre 7%^(5)^ e 23%^(5)^. Entretanto, no ambiente de terapia intensiva, a ocorrência se eleva para 39%, com a mortalidade em torno de 49%^(6)^.

Ao observar o desenvolvimento de IRA no cenário de cuidados intensivos, constataram-se uma maior gravidade dos pacientes, prolongamento do período de internação, maior uso de antibióticos e de drogas vasoativas, e necessidade de ventilação mecânica invasiva (VMI)^(7)^.

Nesse contexto, diversos escores prognósticos têm sido desenvolvidos e utilizados na terapia intensiva, abrangendo uma variedade de características clínicas e dados laboratoriais, com objetivo de avaliar a gravidade dos pacientes e direcionar medidas e intervenção. Os escores *Simplified Acute Physiology Score* 3 (SAPS 3), *Sequential Organ Failure Assessment* (SOFA) e *Nursing Activities Score* (NAS) têm sido utilizados para avaliação prognóstica de gravidade. Eles incorporam diferentes variáveis clínicas, laboratoriais e fisiológicas para fornecer uma estimativa da gravidade da doença, o risco de mortalidade e a carga de trabalho da enfermagem de pacientes críticos^(8)^. A VMI é um tipo de suporte ventilatório frequente na UTI, no qual há substituição total ou parcial da ventilação espontânea. No Brasil, cerca de 36,2% dos pacientes críticos estão em ventilação mecânica por diferentes etiologias^(9)^. A mortalidade de pacientes em ventilação mecânica tende a aumentar à medida que ocorre a falência de múltiplos órgãos, incluindo dos rins^(8)^. Esse cenário evidencia as dificuldades significativas no manejo clínico desses pacientes, reforçando a importância de uma avaliação prognóstica precisa e orientada às necessidades específicas de cada paciente^(10)^. No entanto, a magnitude dessa ocorrência em pacientes críticos e idosos ainda é pouco descrita, assim como o impacto da VMI na função renal, o que reforça a importância de melhorar o conhecimento sobre os fatores ventilatórios que podem impactar o funcionamento subótimo da função renal^(11)^.

Embora a associação entre VMI e a ocorrência de IRA em pacientes críticos seja reconhecida, os mecanismos que sustentam essa interação ainda não estão totalmente elucidados, especialmente no que diz respeito à influência de diferentes parâmetros ventilatórios sobre a perfusão renal. Portanto, o presente estudo visa avaliar o impacto do suporte ventilatório mecânico invasivo na função renal e verificar os fatores predisponentes para o desenvolvimento de IRA em relação à utilização de VMI em terapia intensiva.

## MÉTODO

### Desenho, Período e Local do Estudo

Trata-se de estudo observacional de coorte^(12)^, retrospectivo de abordagem quantitativa^(12)^, referente ao período de janeiro a dezembro de 2023, baseado nas diretrizes do *STrengthening the Reporting of OBservational studies in Epidemiology* ^(13)^. Desenvolvido em uma UTI mista (clínica e coronariana), constituída de 19 leitos cirúrgicos e clínicos de um hospital de ensino terciário localizado na região Centro-Oeste do Brasil.

### Amostra e Critérios de Inclusão

A população foi composta de pacientes clínicos e cirúrgicos internados na UTI. A amostra foi não probabilística, de conveniência e constituída de 51 pacientes, conforme detalhado no fluxograma (Figura 1). Foram incluídos pacientes com idade ≥ 18 anos com alterações sustentadas da CrS ≥ 0,3 mg/dL em relação ao valor basal por pelo menos pelo menos 48 horas, conforme classificação do KDIGO^(2,4)^, além de pacientes em regime de internação na especialidade geral ou coronariana submetidos à VMI por pelo menos 48 horas.

**Figura 1:** Fluxograma referente aos critérios de seleção dos participantes.

Foram excluídos pacientes que internaram mais de uma vez, com taxa de filtração glomerular estimada (TFGe), conforme *Chronic Kidney Disease Epidemiology Collaboration* **(**CKD-Epi), menor que 30 mL/min, sem dosagem da CrS, com tempo de permanência na UTI inferior a 48 horas, e em cuidados paliativos e/ou transplantados renais, pacientes em programa crônico de diálise.

### Procedimento de Coleta de Dados

Os dados foram coletados por meio de instrumento elaborado no formato de questionário estruturado, com itens fechados, subdividido em cinco domínios constituídos pelas seguintes variáveis: identificação (peso, etnia, altura, idade, sexo); desfecho; história pregressa (comorbidades); história atual (função renal, dados laboratoriais, VMI, modo ventilatório, pressão expiratória final positiva (PEEP), fração inspirada de oxigênio, volume corrente, frequência respiratória, drogas vasoativas/antibióticos na UTI); e escores (NAS, SAPS 3 e SOFA).

A dosagem de creatinina foi realizada pelo método de reação cinética com tampão de Jaffé sem desproteinização, e a coleta da CrS foi conduzida pelos profissionais de acordo com a rotina da unidade.

Consulta e extração dos dados do prontuário do paciente, para registro das variáveis do estudo no questionário, e o posterior armazenamento em banco de dados elaborado no *Microsoft Office Professional Plus 2019* foram realizados por pares, dois pesquisadores independentes, visando mitigar a ocorrência de vieses e aumentar a confiabilidade dos resultados.

No acompanhamento diário dos dados laboratoriais e clínicos dos pacientes em VMI, durante 15 dias consecutivos e, posteriormente, ao término da internação na UTI, verificou-se a ocorrência de IRA e demais desdobramentos clínicos. Idoso foi o indivíduo com idade ≥ 60 anos^(14)^.

Alguns conceitos foram assumidos para padronização da coleta de dados. Os escores prognósticos adotados seguiram algumas fontes de dados, como o SAPS 3, definido como um instrumento composto por parâmetros tangentes a um escore fisiológico agudo e prévio capaz de gerar um prognóstico para gravidade e risco de óbito em pacientes que estão sob cuidados em UTIs^(15)^. O SOFA representa um escore capaz de estimar o risco de mortalidade a partir da avaliação contínua de disfunções orgânicas em níveis fisiológicos e laboratoriais^(16)^.

Para avaliação da carga de trabalho da equipe de enfermagem, foi utilizado o NAS como instrumento capaz de avaliar a carga de trabalho do profissional enfermeiro em ambiente de terapia intensiva, propondo interpretações sobre dimensionamento de equipe e manutenção de cuidados específicos^(17)^.

A VMI foi definida como suporte ventilatório, o qual, por meio de tubo endotraqueal ou cânula de traqueostomia, promove substituição total ou parcial da ventilação espontânea^(18)^, sendo a ventilação mecânica prolongada considerada como suporte ventilatório por período superior a sete dias^(19)^.

Os casos de VMI foram identificados com base no dispositivo de oxigênio registrado como “traqueostomia, ventilação mecânica” ou “tubo endotraqueal” no prontuário do paciente em qualquer momento da internação na UTI. A duração da VMI foi calculada como a soma dos dias durante os quais um paciente foi registrado com necessidade de VMI, conforme definido acima.

A avaliação da função renal foi baseada no critério de CrS estabelecido nas diretrizes do KDIGO, devido à ausência de um registro sistemático e preciso do volume urinário, que classifica a gravidade da IRA nos seguintes estágios: KDIGO 1 – valor de CrS maior ou igual a 0,3 mg/dL; KDIGO 2 – aumento de 2,0 a 2,9 vezes no nível de CrS em comparação com o nível basal; KDIGO 3 – aumento de 3 vezes no nível de CrS em comparação com o nível basal, valor de creatinina ≥ 4 mg/dL ou início de terapia de substituição renal^(5)^.

O nível basal de CrS foi o valor mais baixo no período de 365 dias mais recente antes da internação por IRA, ou o valor mais baixo nos primeiros sete dias antes da internação^(20)^. As TFGe foram calculadas pela CKD-Epi^(4)^.

A recuperação da função renal foi calculada nos pacientes com IRA por meio da razão da CrS no momento da avaliação em relação à CrS basal. Há diferentes níveis de recuperação. Neste estudo, foi avaliada a recuperação total da função renal: quando a creatinina retorna ao valor da CrS basal^(20)^.

Já a doença renal aguda (DRA) foi identificada quando se constatou IRA persistente por um período entre sete a 90 dias após exposição a algum evento agressor^(20)^.

### Análise Estatística

Antes da análise de dados, a normalidade dos dados contínuos foi testada por meio do teste de Kolmogorov-Smirnov. Foi constatada amostra assimétrica. Dessa forma, os dados foram expressos como mediana (intervalo interquartil), e a diferença entre grupos foi comparada pelo teste U de Mann-Whitney para amostras independentes. As variáveis categóricas foram expressas em números (porcentagem) e comparadas pelo teste qui-quadrado ou teste de Fisher.

A análise estatística foi conduzida pelo *software Statistical Package for the Social Sciences* 23.0. Todos os testes foram bicaudais, e *p* < 0,05 foi considerado significância estatística.

### Aspectos Éticos

O estudo foi conduzido de acordo com as diretrizes de ética nacionais e internacionais. Foi aprovado pelo Comitê de Ética em Pesquisa com Seres Humanos da Faculdade de Ciências e Tecnologias em Saúde da Universidade de Brasília, sob Certificado de Apresentação para Apreciação Ética n° 61486022.0.0000.8093 e Parecer n° 7.165.243. Todos os pacientes incluídos assinaram o Termo de Consentimento Livre e Esclarecido.

## RESULTADOS

Foram acompanhados 51 pacientes do sexo masculino, pardos e em VMI. A idade mediana foi de 61 anos, sendo predominante nos grupos com e sem IRA. O sobrepeso foi uma característica dos pacientes sem IRA [27,8 (23,2– 32,7)] kg/m^2^. A hipertensão arterial (45,1%) foi a comorbidade mais frequente entre os pacientes. Predominaram pacientes internados para tratamento clínico (82,4%). Antibióticos carbapenêmicos (52,9%) foram usados comumente, assim como a droga vasoativa mais administrada foi a noradrenalina (80,4%) (Tabela 1). Ressalta-se que 28,9% dos pacientes com IRA necessitaram de terapia de suporte renal. Mais da metade dos pacientes em ventilação mecânica necessitaram de PEEP entre 5 e 10 cm/H_2_O (90,2%). Destaca-se que 50% dos pacientes com IRA permaneceram mais de sete dias em VMI. O óbito no grupo com IRA acometeu mais de 50% dos pacientes. O tempo de internação mediano global foi de 15 dias (Tabela 1).

**Tabela 1:** Caracterização demográfica e clínica - Distrito Federal, Brasília, Brasil, 2023.

Variáveis	Sem IRA (n = 13)	Com IRA (n = 38)	Total (n = 51)
Sexo masculino *n (%)*	9 (69,2)	22 (57,9)	31 (60,8)
Idade (anos) *média ± DP; mediana (IIQ)*	58 ± 17 63 (43–69)	55 ± 18 61 (43–69)	56 ± 18 61 (44–69)
IMC (kg/m^2^) *média ± DP; mediana (IIQ)*	27,6 ± 5,0 27,8 (23,2–32,7)	23,7 ± 6,2 23,4 (18,3–28,7)	24,9 ± 6,0 24,6 (20,8–29,6)
Raça *n (%)*	
Branca	1 (7,7)	5 (13,2)	6 (11,8)
Preta	0 (0,0)	1 (2,6)	1 (2,0)
Parda	10 (76,9)	27 (71,1)	37 (72,5)
Outra	1 (7,7)	0 (0,0)	1 (2,0)
Não declarada	1 (7,7)	5 (13,2)	6 (11,8)
Comorbidades *n (%)*	
Diabetes mellitus	4 (30,8)	8 (21,1)	12 (23,5)
Hipertensão arterial	6 (46,2)	17 (44,7)	23 (45,1)
DPOC	2 (15,4)	6 (15,8)	8 (15,7)
IAM	2 (15,4)	8 (21,1)	10 (19,6)
IRPa	1 (7,7)	8 (21,1)	9 (17,6)
Tabagismo	4 (30,8)	14 (36,8)	18 (35,3)
Etilismo	4 (30,8)	7 (18,4)	11 (21,6)
Motivo de internação *n (%)*	
Clínico	11 (84,6)	31 (81,6)	42 (82,4)
Cirúrgico	2 (15,4)	7 (18,4)	9 (17,6)
Medicações *n (%)*	
Noradrenalina	8 (61,5)	33 (86,8)	41 (80,4)
Vasopressina	4 (30,8)	17 (44,7)	21 (41,2)
Amicacina (aminoglicosídeo)	2 (15,4)	8 (21,1)	10 (19,6)
Vancomicina (glicopeptídeo)	3 (23,1)	17 (44,7)	20 (39,2)
Meropenem (carbapenêmico)	6 (46,2)	21 (55,3)	27 (52,9)
Tazocin (lactamase)	4 (30,8)	13 (34,2)	17 (33,3)
TSR *n (%)*	0 (0,0)	11 (28,9)	11 (21,6)
PEEP *n (%)*	
Grupo 1 (maior PEEP <= 5)	0 (0,0)	3 (7,9)	3 (5,9)
Grupo 2 (5< maior PEEP <= 10)	13 (100,0)	33 (86,8)	46 (90,2)
Grupo 3 (maior PEEP > 10)	0 (0,0)	2 (5,3)	2 (3,9)
Desfecho da UTI *n (%)*	
Óbito	5 (38,5)	22 (57,9)	27 (52,9)
Alta clínica	0 (0,0)	3 (7,9)	3 (5,9)
Alta para casa	1 (7,7)	1 (2,6)	2 (3,9)
Encaminhado para outra unidade hospitalar	7 (53,8)	12 (31,6)	19 (37,3)
Modo ventilatório *n (%)*	
Assistido controlado	15 (115,4)	44 (115,8)	59 (115,7)
Pressão suporte	10 (76,9)	27 (71,1)	37 (72,5)
Tempo de VMI (>sete dias) *n (%)*	4 (30,8)	19 (50,0)	23 (45,1)
Tempo de internação *média ± DP; mediana* (IIQ)	22 ± 16 18 (8–34)	19 ± 14 15 (8–29)	20 ± 14 15 (8–30)

Legenda: DP – desvio padrão; IIQ – intervalo interquartílico; IMC – Índice de Massa Corporal; DPOC – doença pulmonar obstrutiva crônica; IAM – infarto agudo do miocárdio; IRPa – insuficiência respiratória aguda; TSR – terapia de suporte renal; PEEP – pressão expiratória final positiva; VMI – ventilação mecânica invasiva; UTI – Unidade de Terapia Intensiva; IRA – injúria renal aguda.

Pacientes com IRA apresentaram alteração significativa dos níveis séricos do lactato (valor de p = 0,04), observandose SOFA e NAS significativamente elevados no grupo com IRA em VMI (valor de p = 0,003 *versus* 0,02). Os níveis de hemoglobina de forma geral se mostraram reduzidos (valor de p = 0,02). SAPS 3 e o SOFA, durante internação na UTI, mantiveram-se elevados, representando risco elevado de mortalidade (Tabela 2).

**Tabela 2:** Caracterização da gravidade clínica e laboratorial – Distrito Federal, Brasília, Brasil, 2023.

Variáveis	Sem IRA (n = 13)	Com IRA (n = 38)	Total (n = 51)	Valor de p
Lactato médio (mg/dL) *média ± DP; mediana (IIQ)*	23,9 ± 27,5 13 (11–15)	26,5 ± 24,1 16 (13–24)	25,9 ± 24,7 15 (13–24)	0,04
Hb médio (%) *média ± DP; mediana (IIQ)*	4,8 ± 2,2 4,7 (3,2–6,3)	6,7 ± 2,5 6,4 (5,3–8,2)	6,2 ± 2,5 6,1 (4,7–8,1)	0,02
PEEP (cm/H_2_O) máximo *média ± DP; mediana (IIQ)*	8,4 ± 1,5 8 (8–10)	8,0 ± 1,8 8 (6–10)	8,1 ± 1,8 8 (7–10)	0,4
SAPS 3 média ± DP; *mediana (IIQ)*	69 ± 15 74 (58–83)	68 ± 13 69 (62–75)	68 ± 13 69 (59–75)	0,6
NAS máximo *média ± DP; mediana (IIQ)*	105,7 ± 6,4 107,3 (104,5–107,3)	113,3 ± 11,1 111,2 (107,3–115,0)	112 ± 11 108,4 (107,3–115,0)	0,02
SOFA máximo *média ± DP; mediana (IIQ)*	6,3 ± 2,3 6,0 (4,5–7,5)	9,0 ± 2,6 9,0 (7,5–11,0)	8,3 ± 2,8 8 (6–10)	0,003

Legenda: DP – desvio padrão; IIQ – intervalo interquartílico; Hb – hemoglobina; PEEP – pressão expiratória final positiva; SAPS 3 – *Simplified Acute Physiology Score* 3; NAS – *Nursing Activities Score*; SOFA – *Sequential Organ Failure Assessment*; IRA – injúria renal aguda.

Os resultados mostram que a maioria dos pacientes (41,2%) evoluiu com IRA de maior gravidade (KDIGO 3), sendo que 33,3% dos pacientes evoluíram com comprometimento leve e moderado da função renal (KDIGO 1 e KDIGO 2) (Tabela 3).

**Tabela 3:** Distribuição dos pacientes conforme a gravidade do comprometimento renal – Distrito Federal, Brasília, Brasil, 2023.

KDIGO	Critério creatinine n (%)
Sem alteração renal	13 (25,5%)
KDIGO 1	8 (15,7%)
KDIGO 2	9 (17,6%)
KDIGO 3	21 (41,2%)

Legenda: KDIGO – *Kidney Disease Improving Global Outcome.*

Ressalta-se que sete (18,8%) tiveram DRA caracterizado por IRA persistente por mais de sete dias. Do total de 38 pacientes com IRA, 21 (55,3%) pacientes recuperaram a função renal até o 15º dia de acompanhamento.

Observa-se na Tabela 4 que tempo de VMI não influenciou de forma significativa a ocorrência de óbitos e IRA. Os resultados mostram que não houve diferença significativa entre as pontuações dos escores SOFA, SAPS 3 e NAS, independentemente do tempo de uso da VMI. A PEEP máxima foi significativamente maior no grupo que permaneceu em VMI prolongada (superior a sete dias) (valor de p = 0,04) e maior tempo de internação na UTI (valor de p = 0,005).

**Tabela 4:** Associação das variáveis tempo de ventilação mecânica invasiva e desfecho – Distrito Federal, Brasília, Brasil, 2023

Variáveis	Tempo de VMI <= sete dias (n = 28)	Tempo de VMI > sete dias (n = 23)	Valor de p
Óbito *n (%)*	14 (50,0)	13 (56,5)	0,6
Lactato médio (mg/dL)	15 (53,6)	3 (13,0)	0,002
pH *(mediana (IIQ))*	7,39 (7,31–7,43)	7,42 (7,37–7,45)	0,13
IRA *n (%)*	19 (67,9)	19 (82,6)	0,2
IRA KDIGO 2 ou KDIGO 3 *n (%)*	14 (50,0)	16 (69,6)	0,2
PEEP máxima *média ± DP; mediana (IIQ)*	7,7 ± 1,8 8 (6–9)	8,6 ± 1,5 8 (8–10)	0,04
SAPS3 *média ± DP; mediana (IIQ)*	69 ± 16 69 (55–79)	68 ± 9 69 (64–74)	0,8
NAS máximo *média ± DP; mediana (IIQ)*	110,9 ± 10,7 107,6 (106,7–115,0)	112,8 ± 11,0 109,1 (107,3–115,0)	0,4
SOFA máximo *média ± DP; mediana (IIQ)*	7,8 ± 2,9 8 (5–10)	9,0 ± 2,6 9 (7–10)	0,2
Tempo de internação *média ± DP; mediana (IIQ)*	16 ± 14 10 (7–24)	24 ± 14 20 (15–34)	0,005

Legenda: DP – desvio padrão; IIQ – intervalo interquartílico; VMI – ventilação mecânica invasiva; IRA – injúria renal aguda; PEEP – pressão expiratória final positiva; SAPS 3 – *Simplified Acute Physiology Score* 3; KDIGO – *Kidney Disease Improving Global Outcome*; NAS – *Nursing Activities Score*; SOFA – *Sequential Organ Failure Assessment*.

## DISCUSSÃO

Observou-se maior prevalência de VMI em pacientes críticos do sexo masculino com IRA, especialmente nos casos mais graves, classificados como KDIGO 3. O suporte ventilatório invasivo foi mais frequente entre idosos, refletindo maior gravidade clínica, conforme evidenciado pelo SAPS 3 e SOFA, e indicando maior risco de mortalidade. Além disso, o tempo de ventilação superior a sete dias se associou a internações prolongadas e à necessidade de maiores níveis de PEEP.

O SOFA e o SAPS 3 são amplamente utilizados para avaliar gravidade e risco de mortalidade em pacientes críticos, e têm demonstrado capacidade prognóstica aceitável para predizer o desenvolvimento de IRA^(21,22)^. Essa associação decorre do fato de que escores mais elevados refletem maior disfunção orgânica, resposta inflamatória exacerbada, estresse oxidativo e redução da perfusão renal^(23)^. Paralelamente, o NAS também se mostrou elevado, o que indica maior sobrecarga de trabalho da equipe de enfermagem, especialmente em pacientes com IRA. Esse achado, observado em nosso estudo, pode estar relacionado à necessidade de uso de drogas vasoativas, alterações bioquímicas, tempo prolongado de internação e suporte ventilatório invasivo, que demandam maior tempo de monitorização e cuidados intensivos contínuos^(22)^.

Apesar de uso onipresente da VMI como uma tecnologia que salva vidas, à medida que sua duração se estende, o risco de complicações e mortalidade aumenta. Em nosso estudo, pacientes com maior tempo de VMI evoluíram mais frequentemente com IRA. O óbito foi constatado em mais da metade dos pacientes em VMI com e sem IRA, o que mostra a importância de priorizar o início dos processos de cuidado para o desmame dos pacientes da VMI o mais rápido possível^(19)^.

Consoante a tais resultados, estudo anterior demonstrou que existe uma interação inflamatória entre o pulmão e o rim, de modo que a lesão em um órgão contribui para a lesão e inflamação no outro. Portanto, os pacientes com IRA podem ser mais predispostos à lesão pulmonar induzida pelo ventilador mecânico, sendo, assim, um dos principais contribuintes para a falência de múltiplos órgãos e morte em pacientes gravemente doentes^(23)^. Em nosso estudo, maior tempo de ventilação mecânica impactou no tempo de internação prolongado (p = 0,005).

A duração da VMI e o tempo de internação na UTI podem ser considerados indicadores, pelo menos parciais, da qualidade do atendimento. Foi relatado que 5 a 20% dos pacientes de UTI necessitam de ventilação mecânica por mais de sete dias. Entre pacientes gravemente enfermos, o tempo médio de internação na UTI varia de 2 a 13 dias, dependendo do perfil do paciente e da gravidade do caso. Neste estudo, o tempo mediano de internação dos pacientes em VMI com e sem IRA foi de 15 dias. Estudo multicêntrico realizado no Brasil mostrou que pacientes em VMI tiveram um tempo médio de internação na UTI de dez dias, com alta mortalidade hospitalar (42%)^(24)^. Em nosso estudo, o óbito por qualquer causa acometeu mais de 50% dos pacientes em VMI.

Apesar dos prejuízos da VMI na função renal, além de outros fatores como hemoglobina reduzida e alteração de lactato identificados na atual investigação, a recuperação renal foi reconhecida em mais da metade dos 74,5% pacientes acometidos pela IRA até o 15º dia de acompanhamento, condição esperada após tratamento adequado, ainda que aproximadamente um em cada cinco dos pacientes com IRA tenha evoluído com DRA, ou seja, quando há persistência da IRA por mais de sete dias^(20)^. Um fator que pode ter contribuído foi a elevação significativa do lactato. No contexto de pacientes gravemente enfermos, como nos subgrupos com IRA, níveis elevados de lactato frequentemente significam hipoperfusão tecidual e oxigenação insuficiente, predispondo à disfunção renal^(10)^. Além disso, a anemia também pode se revelar como fator que pode aumentar a chance de mortalidade, condições identificadas entre os pacientes do nosso estudo. Deve ser valorizado que quadros de anemia também podem resultar em incompatibilidade no fornecimento e consumo de oxigênio, particularmente em órgãos que dependem principalmente de metabolismos oxidativos, como as células epiteliais tubulares renais, favorecendo a ocorrência da IRA em pacientes gravemente enfermos^(25)^.

Estudos atuais mostraram que o rim é um importante órgão gliconeogênico, no qual o lactato é o substrato primário da gliconeogênese renal em condições fisiológicas. Durante a IRA, alterações na glicólise e na gliconeogênese nos rins perturbam significativamente o equilíbrio metabólico do lactato, o que exerce impactos na gravidade e no prognóstico da IRA, e pode inclusive contribuir para maior mortalidade nesse grupo em relação ao grupo sem IRA^(24,26)^.

Nessa vertente, o emprego de modelos de predição clínica para detectar o início de IRA grave na primeira semana de internação na UTI durante o início da VMI pode ser uma alternativa promissora para detecção precoce, visto a frequente ocorrência de IRA em pacientes predominantemente mais velhos e o agravo prognóstico dos pacientes expressos por alteração do SAPS 3 e SOFA^(22)^, como verificado em nossa investigação.

No presente estudo, a maioria dos pacientes necessitou de noradrenalina e vasopressina, potentes vasoconstritores, e quase 90% dos pacientes com IRA necessitaram de PEEP maior que cinco e menor ou igual a 10 cmH_2_O. A PEEP mais elevada foi encontrada com mais frequência em pacientes com necessidade mais prolongada de VMI, condição também evidenciada em coorte retrospectiva de pacientes admitidos em UTI com COVID-19^(27)^.

De forma geral, a ventilação mecânica pode provocar distúrbios hemodinâmicos importantes, especialmente pela ação da PEEP, que eleva a pressão intratorácica, reduz o retorno venoso e, consequentemente, o débito cardíaco. Estudos, como o de Ottolina *et al*., demonstraram que o uso de PEEP elevada pode aumentar em até cinco vezes o risco de desenvolvimento de IRA. Embora a associação entre ventilação e IRA seja bem documentada, o mecanismo exato ainda não está totalmente esclarecido. Entre as hipóteses mais aceitas, estão a redução da perfusão glomerular, a diminuição do fluxo sanguíneo renal e a ativação de mediadores inflamatórios sistêmicos, que, em conjunto, contribuem para a lesão renal^(27)^.

Ressalta-se também que o uso de doses mais elevadas de vasopressores pode estar associado tanto ao aumento da resposta inflamatória quanto aos efeitos hemodinâmicos da ventilação com pressão positiva em níveis mais altos de PEEP. Valores elevados de PEEP podem comprometer a perfusão renal e a TFGe de forma mais acentuada, favorecendo o desenvolvimento de disfunção renal. Em nossa análise, a maioria dos pacientes utilizou PEEP máxima de 10 cmH_2_O, e apenas uma parcela menor necessitou de valores superiores. Estudos experimentais ressaltam que, embora os efeitos adversos da PEEP sejam mais evidentes no sistema respiratório, também podem ocorrer repercussões extrapulmonares significativas, incluindo prejuízos à função renal^(27,28)^. Uma vez instalada a IRA, a atuação colaborativa da equipe interprofissional, em especial da enfermagem, deve se concentrar em prevenir novas lesões, evitar o uso de medicamentos nefrotóxicos e assegurar a dosagem adequada das terapias farmacológicas^(29)^.

Nessa vertente, meta-análise destaca que o reconhecimento precoce da IRA pode favorecer a sua reversão ou, ainda, a recuperação da função renal, contribuindo para que os pacientes recebam fluidos e medicamentos adequados, prevenindo, assim, o agravamento da função renal ou qualquer possibilidade de toxicidade. Pacotes de cuidados para IRA permitem que não nefro-logistas tomem medidas rápidas para prevenir e tratar IRA^(30)^.

Pacientes internados, de maior gravidade, como em nosso estudo, possuem maior risco de desenvolver IRA, o que está associado à alta mortalidade. As diretrizes de consenso para IRA recomendam tratamento imediato, incluindo a manutenção da pressão de perfusão, melhora do estado de fluidos, prevenção de nefrotoxinas e prevenção de hiperglicemia^(30)^.

A aplicabilidade e eficácia de escores prognósticos em pacientes gravemente enfermos com IRA podem variar devido às características dessa população, como a presença de múltiplas comorbidades, instabilidade hemodinâmica e exposição a terapias intensivas. No atual estudo, abordamos SAPS 3, SOFA e NAS, sendo este último referente à carga de trabalho da equipe de enfermagem. Apesar de não mostrarem relação significativa com o tempo de VMI, evidenciaram relação significativa com a IRA como em estudos de coorte prospectivo brasileiro^(22,31)^. Tem-se verificado que o poder discriminatório do NAS indica alta demanda de cuidados pela equipe de enfermagem, e o maior valor do NAS tem sido associado, inclusive, ao desenvolvimento de IRA em pacientes críticos^(21,22)^.

Nosso estudo possui limitações, como coorte de centro único, e a generalização dos nossos resultados é limitada, com presença de risco de viés de aferição e pequeno tamanho amostral.

Os achados deste estudo contribuem significativamente para o fortalecimento da prática de enfermagem em UTIs, ao evidenciar a associação entre a VMI e a ocorrência de IRA, especialmente em estágios mais graves da classificação KDIGO. Esse conhecimento reforça a importância da vigilância clínica contínua realizada pela equipe de enfermagem diante de alterações hemodinâmicas e marcadores laboratoriais de função renal em pacientes críticos em suporte ventilatório.

Além disso, os resultados destacam a necessidade de implementação de protocolos de enfermagem que integrem o monitoramento precoce da função renal aos cuidados respiratórios, promovendo intervenções oportunas que possam minimizar a progressão da IRA. Tais práticas ampliam o escopo da atuação do enfermeiro intensivista, favorecendo decisões clínicas baseadas em evidências, com impacto direto na segurança e nos desfechos dos pacientes.

## CONCLUSÃO

O impacto da ventilação mecânica em pacientes críticos foi evidenciado pela maior prevalência de IRA grave (KDIGO 3) e DRA. O suporte ventilatório invasivo foi mais prevalente entre os idosos, evidenciando a gravidade dos pacientes a partir dos SAPS 3 e SOFA e consequente maior risco de morte. Alguns fatores, como as alterações bioquímicas do lactato e hemoglobina, mostraram-se significativas entre os pacientes em suporte ventilatório mecânico invasivo com IRA.

O tempo de ventilação mecânica não repercutiu necessariamente em maior carga de trabalho para a equipe de enfermagem, haja vista que, por si só, o cuidado de pacientes com IRA impactou a elevação do NAS, independentemente do tempo que permaneceram em ventilação mecânica, destacando que a vigilância permanente pela equipe de enfermagem é fundamental para detecção precoce de alterações hemodinâmicas e dos marcadores laboratoriais de função renal em pacientes mais graves sob suporte ventilatório. O desenvolvimento e a implementação de protocolos são essenciais, permitindo intervenções apropriadas que podem minimizar a progressão da IRA, melhorar os desfechos clínicos e aumentar a segurança do paciente.

## Data Availability

Todo o conjunto de dados que dá suporte aos resultados deste estudo foi publicado no próprio artigo.

# Translation: Impacto del soporte ventilatorio mecánico invasivo sobre la función renal en pacientes críticos

**DOI:** 10.1590/1980-220X-REEUSP-2025-0309es

**Authors:** João Marcos Santos Rocha, Tayse Tâmara da Paixão Duarte, Michelle Zampieri Ipolito, Walterlânia Silva Santos, Paulo Percio Mota Magro, Marcia Cristina da Silva Magro

**Affiliations:** 1 Universidade de Brasília, Faculdade de Ciências e Tecnologias em Saúde, Brasília, DF, Brasil.; 2 Instituto Federal de Brasília, Brasília, DF, Brasil.

**Keywords:** Lesión Renal Aguda, Respiración Artificial, Unidades de Cuidados Intensivos, Puntuaciones en la Disfunción de Órganos

## Abstract

**Objetivo:**

Evaluar el impacto de la ventilación mecánica invasiva en la función renal y verificar los factores predisponentes para el desarrollo de lesión renal aguda en relación con el uso de ventilación mecánica invasiva en cuidados intensivos.

**Método:**

Estudio observacional, retrospectivo y cuantitativo de cohorte. La muestra fue no probabilística, de conveniencia, y consistió en 51 pacientes. La gravedad de los pacientes se evaluó mediante la Simplified Acute Physiology Score y Sequential Organ Failure Assessment. Todas las pruebas fueron bilaterales, y un valor de p < 0,05 se consideró estadísticamente significativo.

**Resultados:**

Entre los pacientes con lesión renal que recibían ventilación mecánica invasiva, el 41,2% presentó lesión renal grave (estadio 3 de los Kidney Disease Improving Global Outcome). La duración de la ventilación mecánica fue mayor en los pacientes con lesión renal aguda que en aquellos sin insuficiencia renal (19 días frente a cuatro).

**Conclusión:**

El impacto de la ventilación mecánica en pacientes críticos se evidenció por la mayor prevalencia de lesión renal aguda grave. El soporte ventilatorio invasivo fue más frecuente en los ancianos, lo que resaltó la gravedad de los pacientes según las puntuaciones de la Simplified Acute Physiology Score y Sequential Organ Failure Assessment, y, en consecuencia, un mayor riesgo de muerte.

## INTRODUCCIÓN

La lesión renal aguda (LRA) es un síndrome clínico complejo que influye significativamente en el proceso de la enfermedad, empeorando la evolución de un gran número de pacientes hospitalizados, lo que repercute en el aumento de la morbimortalidad y prolonga la estancia hospitalaria^(1)^.

En los países de ingresos bajos y medios, las infecciones y el choque hipovolémico son las causas predominantes de la LRA^(2)^. En los países de ingresos altos, la LRA se presenta principalmente en pacientes de edad avanzada hospitalizados y está relacionada con la sepsis, los medicamentos o los procedimientos invasivos^(2)^. Desde esta perspectiva, la LRA grave se ha mostrado prevalente en las Unidades de Terapia Intensiva (UTI), con una mayor incidencia a lo largo de las últimas décadas en pacientes de edad avanzada, lo que contribuye a una alta mortalidad y da lugar a elevados costos relacionados con el manejo de la LRA, difícil de prevenir^(3)^.

Las directrices de práctica clínica de la Kidney Disease Improving Global Outcome (KDIGO) describen la LRA como un aumento de la creatinina sérica (CrS) ≥ 0,3 mg/dl en dos días o un aumento de la CrS ≥ 1,5 veces con respecto al valor basal o una diuresis < 0,5 ml/kg/h en seis horas^(4)^. La tasa de incidencia de la LRA en pacientes hospitalizados varía entre el 7%^(5)^ y el 23%^(5)^. Sin embargo, en el entorno de cuidados intensivos, la incidencia se eleva al 39%, con una mortalidad en torno al 49%^(6)^.

Al observar el desarrollo de la LRA en el contexto de los cuidados intensivos, se constató una mayor gravedad de los pacientes, una prolongación del período de hospitalización, un mayor uso de antibióticos y fármacos vasoactivos, y la necesidad de ventilación mecánica invasiva (VMI)^(7)^.

En este contexto, se han desarrollado y utilizado en terapia intensiva diversas puntuaciones pronósticas que abarcan una variedad de características clínicas y datos de laboratorio, con el objetivo de evaluar la gravedad de los pacientes y orientar las medidas y la intervención. Las puntuaciones Simplified Acute Physiology Score 3 (SAPS 3), Sequential Organ Failure Assessment (SOFA) y Nursing Activities Score (NAS) se han utilizado para la evaluación pronóstica de la gravedad. Incorporan diferentes variables clínicas, de laboratorio y fisiológicas para proporcionar una estimación de la gravedad de la enfermedad, el riesgo de mortalidad y la carga de trabajo de enfermería de los pacientes críticos^(8)^. La VMI es un tipo de soporte ventilatorio frecuente en la UTI, en el que se sustituye total o parcialmente la ventilación espontánea.

En Brasil, alrededor del 36,2% de los pacientes críticos están en ventilación mecánica por diferentes etiologías^(9)^. La mortalidad de los pacientes con ventilación mecánica tiende a aumentar a medida que se produce un fallo multiorgánico, incluyendo los riñones^(8)^. Este escenario pone de manifiesto las importantes dificultades en el manejo clínico de estos pacientes, lo que refuerza la importancia de una evaluación pronóstica precisa y orientada a las necesidades específicas de cada paciente^(10)^. Sin embargo, la magnitud de esta ocurrencia en pacientes críticos y ancianos aún está poco descrita, al igual que el impacto de la VMI en la función renal, lo que refuerza la importancia de mejorar el conocimiento sobre los factores ventilatorios que pueden afectar el funcionamiento subóptimo de la función renal^(11)^. Aunque se reconoce la asociación entre la VMI y la aparición de la LRA en pacientes críticos, los mecanismos que sustentan esta interacción aún no están totalmente esclarecidos, especialmente en lo que respecta a la influencia de diferentes parámetros ventilatorios sobre la perfusión renal. Por lo tanto, el presente estudio tiene como objetivo evaluar el impacto del soporte ventilatorio mecánico invasivo en la función renal y verificar los factores predisponentes para el desarrollo de la LRA en relación con el uso de VMI en terapia intensiva.MÉTODODiseño, período y lugar del estudio Se trata de un estudio observacional de cohorte^(12)^, retrospectivo y de enfoque cuantitativo^(12)^, correspondiente al período de enero a diciembre de 2023, basado en las directrices de STrengthening the Reporting of OBservational studies in Epidemiology^(13)^. Se desarrolló en una UTI mixta (clínica y coronaria), compuesta por 19 camas quirúrgicas y clínicas de un hospital universitario terciario ubicado en la región centro-oeste de Brasil.Muestra y criterios de inclusiónLa población estuvo compuesta por pacientes clínicos y quirúrgicos ingresados en la UTI. La muestra fue no probabilística, de conveniencia y constó de 51 pacientes, como se detalla en el diagrama de flujo (Figura 1). Se incluyeron pacientes ≥ 18 años con alteraciones sostenidas de CrS ≥ 0,3 mg/dL en relación con el valor basal durante al menos 48 horas, según la clasificación de la KDIGO(2,4), además de pacientes ingresados en la especialidad general o coronaria sometidos a VMI durante al menos 48 horas. Se excluyó a los pacientes que ingresaron más de una vez, con una tasa de filtración glomerular estimada (TFGe), según la Chronic Kidney Disease Epidemiology Collaboration (CKD-Epi), inferior a 30 mL/min, sin dosificación de CrS, con una estancia en la UTI inferior a 48 horas, y en cuidados paliativos y/o trasplantados renales, pacientes en programa de diálisis crónica.

**Figura 1:** Flujograma relativo a los criterios de selección de los participantes.

### Procedimiento de Recolección de Datos

Los datos se recolectaron mediante un instrumento elaborado en formato de cuestionario estructurado, con preguntas cerradas, subdividido en cinco ámbitos constituidos por las siguientes variables: identificación (peso, etnia, altura, edad, sexo); resultado; antecedentes (comorbilidades); historial actual (función renal, datos de laboratorio, VMI, modo ventilatorio, presión positiva al final de la espiración (PEEP), fracción inspirada de oxígeno, volumen corriente, frecuencia respiratoria, fármacos vasoactivos/antibióticos en la UTI); y puntuaciones (NAS, SAPS 3 y SOFA).

La dosificación de creatinina se realizó mediante el método de reacción cinética con tampón de Jaffé sin desproteinización, y la recolección de CrS fue realizada por los profesionales de acuerdo con la rutina de la unidad.

La consulta y extracción de los datos de la historia clínica del paciente, para el registro de las variables del estudio en el cuestionario, y el posterior almacenamiento en una base de datos elaborada en *Microsoft Office Professional Plus 2019*, fueron realizadas por pares, dos investigadores independientes, con el fin de mitigar la aparición de sesgos y aumentar la fiabilidad de los resultados.

Em el seguimiento diario de los datos clínicos y de laboratorio de los pacientes en VMI, durante 15 días consecutivos y, posteriormente, al final de la hospitalización en la UTI, verificó la aparición de LRA y otras complicaciones clínicas. Se consideró anciano al individuo con edad ≥ 60 años^(14)^.

Se asumieron algunos conceptos para estandarizar la recopilación de datos. Las puntuaciones pronósticas adoptadas siguieron algunas fuentes de datos, como el SAPS 3, definido como un instrumento compuesto por parámetros tangentes a una puntuación fisiológica aguda y previa capaz de generar un pronóstico de gravedad y riesgo de muerte en pacientes que están bajo cuidados en UTI^(15)^. El SOFA representa una puntuación capaz de estimar el riesgo de mortalidad a partir de la evaluación continua de las disfunciones orgánicas en niveles fisiológicos y de laboratorio^(16)^.

Para evaluar la carga de trabajo del equipo de enfermería, se utilizó el NAS como instrumento capaz de evaluar la carga de trabajo del profesional de enfermería en un entorno de terapia intensiva, proponiendo interpretaciones sobre el dimensionamiento del equipo y el mantenimiento de cuidados específicos^(17)^.

La VMI se definió como soporte ventilatorio que, mediante un tubo endotraqueal o una cánula de traqueotomía, promueve la sustitución total o parcial de la ventilación espontánea^(18)^, considerándose la ventilación mecánica prolongada como soporte ventilatorio por un período superior a siete días^(19)^.

Los casos de VMI se identificaron basándose en el dispositivo de oxígeno registrado como “traqueotomía, ventilación mecánica” o “tubo endotraqueal” en la historia clínica del paciente en cualquier momento de su ingreso en la UTI. La duración de la VMI se calculó como la suma de los días durante los cuales se registró que un paciente necesitaba VMI, tal y como se ha definido anteriormente.

La evaluación de la función renal se basó en el criterio de CrS establecido en las directrices de KDIGO, debido a la ausencia de un registro sistemático y preciso del volumen urinario, que clasifica la gravedad de la LRA en las siguientes etapas: KDIGO 1: valor de CrS mayor o igual a 0,3 mg/dL; KDIGO 2: aumento de 2,0 a 2,9 veces en el nivel de CrS en comparación con el nivel basal; KDIGO 3: aumento de 3 veces en el nivel de CrS en comparación con el nivel basal, valor de creatinina ≥ 4 mg/dL o inicio de terapia de reemplazo renal^(5)^.

El nivel basal de CrS fue el valor más bajo en el período de 365 días más reciente antes de la hospitalización por LRA, o el valor más bajo en los primeros siete días antes de la hospitalización^(20)^. Las TFGe se calcularon mediante CKD-Epi^(4)^.

La recuperación de la función renal se calculó en los pacientes con LRA mediante la relación entre el CrS en el momento de la evaluación y el CrS basal. Existen diferentes niveles de recuperación. En este estudio, se evaluó la recuperación total de la función renal: cuando la creatinina vuelve al valor del CrS basal^(20)^.

Por otro lado, la enfermedad renal aguda (ERA) se identificó cuando se observó LRA persistente durante un período de entre siete y 90 días después de la exposición a algún evento agresor^(20)^.

### Análisis Estadístico

Antes del análisis de los datos, se comprobó la normalidad de los datos continuos mediante la prueba de Kolmogorov-Smirnov. Se observó una muestra asimétrica. De este modo, los datos se expresaron como mediana (intervalo intercuartí-lico) y la diferencia entre los grupos se comparó mediante la prueba U de Mann-Whitney para muestras independientes. Las variables categóricas se expresaron en números (porcentaje) y se compararon mediante la prueba de chi cuadrado o la prueba de Fisher.

El análisis estadístico se realizó con el software Statistical Package for the Social Sciences 23.0. Todas las pruebas fueron bilaterales, y se consideró estadísticamente significativo un valor de p < 0,05.

### Aspectos Éticos

El estudio se llevó a cabo de acuerdo con las directrices éticas nacionales e internacionales. Fue aprobado por el Comité de Ética en Investigación con Seres Humanos de la Facultad de Ciencias y Tecnologías de la Salud de la Universidad de Brasilia, bajo el Certificado de Presentación para Apreciación Ética n.º 61486022.0.0000.8093 y el Dictamen n.º 7.165.243. Todos los pacientes incluidos firmaron el Término de Consentimiento Libre e Informado.

## RESULTADOS

Se realizó un seguimiento de 51 pacientes varones, de raza mestiza y en VMI. La mediana de edad fue de 61 años, predominante en los grupos con y sin LRA. El sobrepeso fue una característica de los pacientes sin LRA [27,8 (23,2–32,7)] kg/m^2^. La hipertensión arterial (45,1%) fue la comorbilidad más frecuente entre los pacientes. Predominaron los pacientes ingresados para tratamiento clínico (82,4%). Se utilizaron comúnmente antibióticos carbapenémicos (52,9%), y el fármaco vasoactivo más administrado fue la noradrenalina (80,4%) (Tabla 1). Cabe destacar que el 28,9% de los pacientes con LRA necesitaron terapia de soporte renal. Más de la mitad de los pacientes con ventilación mecánica necesitaron una PEEP entre 5 y 10 cm/H_2_O (90,2%). Cabe destacar que el 50% de los pacientes con LRA permanecieron más de siete días en VMI. La muerte en el grupo con LRA afectó a más del 50% de los pacientes. La mediana global de la estancia hospitalaria fue de 15 días (Tabla 1).

**Tabla 1:** Caracterización demográfica y clínica – Distrito Federal, Brasilia, Brasil, 2023.

Variables	Sin LRA (n = 13)	Con LRA (n = 38)	Total (n = 51)
Sexo masculino *n (%)*	9 (69,2)	22 (57,9)	31 (60,8)
Edad (años) *media ± DE; mediana (IIC)*	58 ± 17 63 (43–69)	55 ± 18 61 (43–69)	56 ± 18 61 (44–69)
IMC (kg/m^2^) *media ± DE; mediana (IIC)*	27,6 ± 5,0 27,8 (23,2–32,7)	23,7 ± 6,2 23,4 (18,3–28,7)	24,9 ± 6,0 24,6 (20,8–29,6)
Raza *n (%)*			
Blanca	1 (7,7)	5 (13,2)	6 (11,8)
Negra	0 (0,0)	1 (2,6)	1 (2,0)
Moreno	10 (76,9)	27 (71,1)	37 (72,5)
Otra	1 (7,7)	0 (0,0)	1 (2,0)
No declarada	1 (7,7)	5 (13,2)	6 (11,8)
Comorbilidades *n (%)*			
Diabetes mellitus	4 (30,8)	8 (21,1)	12 (23,5)
Hipertensión arterial	6 (46,2)	17 (44,7)	23 (45,1)
EPOC	2 (15,4)	6 (15,8)	8 (15,7)
IAM	2 (15,4)	8 (21,1)	10 (19,6)
IRPa	1 (7,7)	8 (21,1)	9 (17,6)
Tabaquismo	4 (30,8)	14 (36,8)	18 (35,3)
Alcoholismo	4 (30,8)	7 (18,4)	11 (21,6)
Motivo de la hospitalización *n (%)*			
Clínico	11 (84,6)	31 (81,6)	42 (82,4)
Quirúrgico	2 (15,4)	7 (18,4)	9 (17,6)
Medicamentos *n (%)*			
Noradrenalina	8 (61,5)	33 (86,8)	41 (80,4)
Vasopresina	4 (30,8)	17 (44,7)	21 (41,2)
Amicacina (aminoglucósido)	2 (15,4)	8 (21,1)	10 (19,6)
Vancomicina (glucopéptido)	3 (23,1)	17 (44,7)	20 (39,2)
Meropenem (carbapenémico)	6 (46,2)	21 (55,3)	27 (52,9)
Tazocin (lactamasa)	4 (30,8)	13 (34,2)	17 (33,3)
TSR *n (%)*	0 (0,0)	11 (28,9)	11 (21,6)
PEEP *n (%)*			
Grupo 1 (PEEP mayor <= 5)	0 (0,0)	3 (7,9)	3 (5,9)
Grupo 2 (5 < mayor PEEP <= 10)	13 (100,0)	33 (86,8)	46 (90,2)
Grupo 3 (mayor PEEP > 10)	0 (0,0)	2 (5,3)	2 (3,9)
Resultado de la UTI *n (%)*			
Muerte	5 (38,5)	22 (57,9)	27 (52,9)
Alta clínica	0 (0,0)	3 (7,9)	3 (5,9)
Alta a domicilio	1 (7,7)	1 (2,6)	2 (3,9)
Derivado a otro centro hospitalario	7 (53,8)	12 (31,6)	19 (37,3)
Modo ventilatorio *n (%)*			
Asistido controlado	15 (115,4)	44 (115,8)	59 (115,7)
Presión de soporte	10 (76,9)	27 (71,1)	37 (72,5)
Tiempo de VMI (> siete días) *n (%)*	4 (30,8)	19 (50,0)	23 (45,1)
Tiempo *medio* de hospitalización *± DE; mediana* (IIC)	22 ± 16 18 (8–34)	19 ± 14 15 (8–29)	20 ± 14 15 (8–30)

Leyenda: DE: desviación estándar; IIC: intervalo intercuartílico; IMC: índice de masa corporal; EPOC: enfermedad pulmonar obstructiva crónica; IAM: infarto agudo de miocardio; IRPa: insuficiencia respiratoria aguda; TSR: terapia de soporte renal; PEEP: presión espiratoria final positiva; VMI: ventilación mecánica invasiva; UTI: unidad de terapia intensiva; LRA: lesión renal aguda.

Los pacientes con LRA presentaron alteraciones significativas en los niveles séricos de lactato (valor de p = 0,04), observándose SOFA y NAS significativamente elevados en el grupo con LRA en VMI (valor de p = 0,003 *frente a* 0,02). Los niveles de hemoglobina en general se mostraron reducidos (valor de p = 0,02). El SAPS 3 y el SOFA, durante la hospitalización en la UTI, se mantuvieron elevados, lo que representaba un alto riesgo de mortalidad (Tabla 2).

**Tabla 2:** Caracterización de la gravedad clínica y laboratorial – Distrito Federal, Brasilia, Brasil, 2023.

Variables	Sin LRA (n = 13)	Con LRA (n = 38)	Total (n = 51)	Valor de p
Lactato medio (mg/dL) *media ± DE; mediana (IIC)*	23,9 ± 27,5 13 (11–15)	26,5 ± 24,1 16 (13–24)	25,9 ± 24,7 15 (13–24)	0,04
Hb media (%) *media ± DE; mediana (IIC)*	4,8 ± 2,2 4,7 (3,2–6,3)	6,7 ± 2,5 6,4 (5,3–8,2)	6,2 ± 2,5 6,1 (4,7–8,1)	0,02
PEEP (cm/H_2_O) máximo *media ± DE; mediana (IIC)*	8,4 ± 1,5 8 (8–10)	8,0 ± 1,8 8 (6–10)	8,1 ± 1,8 8 (7–10)	0,4
SAPS 3 *media ± DE; mediana (IIC)*	69 ± 15 74 (58–83)	68 ± 13 69 (62–75)	68 ± 13 69 (59–75)	0,6
NAS máximo *media ± DE; mediana (IIC)*	105,7 ± 6,4 107,3 (104,5–107,3)	113,3 ± 11,1 111,2 (107,3–115,0)	112 ± 11 108,4 (107,3–115,0)	0,02
SOFA máximo *media ± DE; mediana (IIC)*	6,3 ± 2,3 6,0 (4,5–7,5)	9,0 ± 2,6 9,0 (7,5–11,0)	8,3 ± 2,8 8 (6–10)	0,003

Leyenda: DE: desviación estándar; IIC: intervalo intercuartílico; Hb: hemoglobina; PEEP: presión espiratoria final positiva; SAPS 3: *Simplified Acute Physiology Score* 3; NAS: *Nursing Activities Score*; SOFA: *Sequential Organ Failure Assessment*; LRA: lesión renal aguda.

Los resultados muestran que la mayoría de los pacientes (41,2%) evolucionaron con LRA de mayor gravedad (KDIGO 3), mientras que el 33,3% de los pacientes evolucionaron con compromiso leve y moderado de la función renal (KDIGO 1 y KDIGO 2) (Tabla 3).

**Tabla 3:** Distribución de los pacientes según la gravedad del compromiso renal – Distrito Federal, Brasilia, Brasil, 2023.

KDIGO	Criterio de creatinine n (%)
Sin alteración renal	13 (25,5%)
KDIGO 1	8 (15,7%)
KDIGO 2	9 (17,6%)
KDIGO 3	21 (41,2%)

Leyenda: KDIGO: *Kidney Disease Improving Global Outcome (Mejora global de los resultados en la enfermedad renal)*.

Cabe destacar que siete (18,8%) tuvieron DRA caracterizada por LRA persistente durante más de siete días. Del total de 38 pacientes con LRA, 21 (55,3%) recuperaron la función renal hasta el decimoquinto día de seguimiento.

En la tabla 4 se observa que el tiempo de VMI no influyó significativamente en la incidencia de muertes y LRA. Los resultados muestran que no hubo diferencias significativas entre las puntuaciones de SOFA, SAPS 3 y NAS, independientemente del tiempo de uso de la VMI. La PEEP máxima fue significativamente mayor en el grupo que permaneció en VMI prolongada (más de siete días) (valor de p = 0,04) y mayor tiempo de hospitalización en la UTI (valor de p = 0,005).

**Tabla 4:** Asociación de las variables tiempo de ventilación mecánica invasiva y resultado – Distrito Federal, Brasilia, Brasil, 2023.

Variables	Tiempo de VMI <= siete días (n = 28)	Tiempo de VMI > siete días (n = 23)	Valor de p
Muerte *n (%)*	14 (50,0)	13 (56,5)	0,6
Lactato medio (mg/dL)	15 (53,6)	3 (13,0)	0,002
pH *(mediana (IIC))*	7,39 (7,31–7,43)	7,42 (7,37–7,45)	0,13
LRA *n (%)*	19 (67,9)	19 (82,6)	0,2
LRA KDIGO 2 o KDIGO 3 *n (%)*	14 (50,0)	16 (69,6)	0,2
PEEP máxima *media ± DE; mediana (IIC)*	7,7 ± 1,8 8 (6–9)	8,6 ± 1,5 8 (8–10)	0,04
SAPS3 *media ± DE; mediana (IIC)*	69 ± 16 69 (55–79)	68 ± 9 69 (64–74)	0,8
NAS máximo *media ± DE; mediana (IIC)*	110,9 ± 10,7 107,6 (106,7–115,0)	112,8 ± 11,0 109,1 (107,3–115,0)	0,4
SOFA máximo *media ± DE; mediana (IIC)*	7,8 ± 2,9 8 (5–10)	9,0 ± 2,6 9 (7–10)	0,2
Tiempo *medio* de hospitalización *± DE; mediana (IIC)*	16 ± 14 10 (7–24)	24 ± 14 20 (15–34)	0,005

Leyenda: DE: desviación estándar; IIC: intervalo intercuartílico; VMI: ventilación mecánica invasiva; LRA: lesión renal aguda; PEEP: presión espiratoria final positiva; SAPS 3: *Simplified Acute Physiology Score* 3; KDIGO: *Kidney Disease Improving Global Outcome*; NAS: *Nursing Activities Score*; SOFA: *Sequential Organ Failure Assessment*.

## DISCUSIÓN

Se observó una mayor prevalencia de VMI en pacientes críticos varones con LRA, especialmente en los casos más graves, clasificados como KDIGO 3. El soporte ventilatorio invasivo fue más frecuente entre los ancianos, lo que refleja una mayor gravedad clínica, según lo evidencian el SAPS 3 y el SOFA, e indica un mayor riesgo de mortalidad. Además, el tiempo de ventilación superior a siete días se asoció con hospitalizaciones prolongadas y la necesidad de mayores niveles de PEEP.

El SOFA y el SAPS 3 se utilizan ampliamente para evaluar la gravedad y el riesgo de mortalidad en pacientes críticos, y han demostrado una capacidad pronóstica aceptable para predecir el desarrollo de LRA^(21,22)^. Esta asociación se debe a que las puntuaciones más altas reflejan una mayor disfunción orgánica, una respuesta inflamatoria exacerbada, estrés oxidativo y una reducción de la perfusión renal^(23)^. Paralelamente, el NAS también se mostró elevado, lo que indica una mayor sobrecarga de trabajo del equipo de enfermería, especialmente en pacientes con LRA. Este hallazgo, observado en nuestro estudio, puede estar relacionado con la necesidad de usar fármacos vasoactivos, alteraciones bioquímicas, tiempo prolongado de hospitalización y soporte ventilatorio invasivo, que requieren más tiempo de monitorización y cuidados intensivos continuos^(22)^.

A pesar del uso omnipresente de la VMI como tecnología que salva vidas, a medida que se prolonga su duración, aumenta el riesgo de complicaciones y mortalidad. En nuestro estudio, los pacientes con mayor tiempo de VMI evolucionaron con mayor frecuencia hacia LRA. Se observó la muerte en más de la mitad de los pacientes en VMI con y sin LRA, lo que demuestra la importancia de priorizar el inicio de los procesos de atención para el destete de los pacientes de la VMI lo más rápido posible^(19)^.

De acuerdo con estos resultados, un estudio anterior demostró que existe una interacción inflamatoria entre el pulmón y el riñón, de modo que la lesión en un órgano contribuye a la lesión e inflamación en el otro. Por lo tanto, los pacientes con LRA pueden ser más propensos a sufrir lesiones pulmonares inducidas por el ventilador mecánico, lo que los convierte en uno de los principales factores que contribuyen al fallo multiorgánico y a la muerte en pacientes gravemente enfermos^(23)^. En nuestro estudio, un mayor tiempo de ventilación mecánica repercutió en una hospitalización prolongada (p = 0,005).

La duración de la VMI y el tiempo de hospitalización en la UTI pueden considerarse indicadores, al menos parciales, de la calidad de la atención. Se ha informado que entre el 5 y el 20% de los pacientes de la UTI necesitan ventilación mecánica durante más de siete días. Entre los pacientes gravemente enfermos, la estancia media en la UTI varía entre 2 y 13 días, dependiendo del perfil del paciente y de la gravedad del caso. En este estudio, la mediana del tiempo de estancia hospitalaria de los pacientes en VMI con y sin LRA fue de 15 días. Un estudio multicéntrico realizado en Brasil mostró que los pacientes en VMI tuvieron una estancia media en la UTI de diez días, con una alta mortalidad hospitalaria (42%)^(24)^. En nuestro estudio, la muerte por cualquier causa afectó a más del 50% de los pacientes en VMI.

A pesar de los daños de la VMI en la función renal, además de otros factores como la reducción de la hemoglobina y la alteración del lactato identificados en la presente investigación, se reconoció la recuperación renal en más de la mitad de los 74,5% de pacientes afectados por LRA hasta el decimoquinto día de seguimiento, condición esperada tras un tratamiento adecuado, aunque aproximadamente uno de cada cinco pacientes con LRA evolucionó con DRA, es decir, cuando hay persistencia de LRA durante más de siete días^(20)^. Un factor que puede haber contribuido fue el aumento significativo del lactato. En el contexto de pacientes gravemente enfermos, como en los subgrupos con LRA, los niveles elevados de lactato suelen significar hipoperfusión tisular y oxigenación insuficiente, lo que predispone a la disfunción renal^(10)^. Además, la anemia también puede revelarse como un factor que puede aumentar la probabilidad de mortalidad, condiciones identificadas entre los pacientes de nuestro estudio. Cabe destacar que los cuadros de anemia también pueden dar lugar a una incompatibilidad entre el suministro y el consumo de oxígeno, especialmente en órganos que dependen principalmente de metabolismos oxidativos, como las células epiteliales tubulares renales, lo que favorece la aparición de LRA en pacientes gravemente enfermos^(25)^.

Estudios recientes han demostrado que el riñón es un importante órgano gluconeogénico, en el que el lactato es el sustrato primario de la gluconeogénesis renal en condiciones fisiológicas. Durante la LRA, los cambios en la glucólisis y la gluconeogénesis en los riñones alteran significativamente el equilibrio metabólico del lactato, lo que repercute en la gravedad y el pronóstico de la LRA, y puede incluso contribuir a una mayor mortalidad en este grupo en comparación con el grupo sin LRA^(24,25,26)^.

En este sentido, el uso de modelos de predicción clínica para detectar el inicio de LRA grave en la primera semana de ingreso en la UTI durante el inicio de la VMI puede ser una alternativa prometedora para la detección precoz, dada la frecuente aparición de LRA en pacientes predominantemente de edad avanzada y el agravamiento del pronóstico de los pacientes expresado por la alteración del SAPS 3 y el SOFA^(22)^, como se ha comprobado en nuestra investigación.

En el presente estudio, la mayoría de los pacientes necesitaron noradrenalina y vasopresina, potentes vasoconstrictores, y casi el 90% de los pacientes con LRA necesitaron una PEEP superior a cinco y menor o igual a 10 cmH_2_O. La PEEP más alta se encontró con mayor frecuencia en pacientes con necesidad más prolongada de VMI, condición también evidenciada en una cohorte retrospectiva de pacientes ingresados en la UTI con COVID-19^(27)^.

En general, la ventilación mecánica puede provocar trastornos hemodinámicos importantes, especialmente por la acción de la PEEP, que eleva la presión intratorácica, reduce el retorno venoso y, en consecuencia, el gasto cardíaco. Estudios como el de Ottolina *et al*. han demostrado que el uso de una PEEP elevada puede aumentar hasta cinco veces el riesgo de desarrollar LRA. Aunque la asociación entre la ventilación y la LRA está bien documentada, el mecanismo exacto aún no está del todo claro. Entre las hipótesis más aceptadas se encuentran la reducción de la perfusión glomerular, la disminución del flujo sanguíneo renal y la activación de mediadores inflamatorios sistémicos, que, en conjunto, contribuyen a la lesión renal^(27)^.

Cabe destacar también que el uso de dosis más altas de vasopresores puede estar asociado tanto al aumento de la respuesta inflamatoria como a los efectos hemodinámicos de la ventilación con presión positiva en niveles más altos de PEEP. Los valores elevados de PEEP pueden comprometer la perfusión renal y la TFGe de forma más acusada, favoreciendo el desarrollo de disfunción renal. En nuestro análisis, la mayoría de los pacientes utilizaron una PEEP máxima de 10 cmH_2_O, y solo una pequeña parte necesitó valores superiores. Los estudios experimentales destacan que, aunque los efectos adversos de la PEEP son más evidentes en el sistema respiratorio, también pueden producirse repercusiones extrapulmonares significativas, incluyendo daños en la función renal^(27,28)^. Una vez instalada la LRA, la actuación colaborativa del equipo interprofesional, en especial del personal de enfermería, debe centrarse en prevenir nuevas lesiones, evitar el uso de medicamentos nefrotóxicos y garantizar la dosificación adecuada de las terapias farmacológicas^(29)^.

En este sentido, un metaanálisis destaca que el reconocimiento precoz de la LRA puede favorecer su reversión o, incluso, la recuperación de la función renal, contribuyendo a que los pacientes reciban los fluidos y medicamentos adecuados, previniendo así el agravamiento de la función renal o cualquier posibilidad de toxicidad. Los paquetes de cuidados para la LRA permiten que los no nefrólogos tomen medidas rápidas para prevenir y tratar la LRA^(30)^.

Los pacientes hospitalizados, de mayor gravedad, como en nuestro estudio, tienen un mayor riesgo de desarrollar LRA, lo que se asocia con una alta mortalidad. Las directrices de consenso para la LRA recomiendan un tratamiento inmediato, que incluye el mantenimiento de la presión de perfusión, la mejora del estado de los fluidos, la prevención de nefrotoxinas y la prevención de la hiperglucemia^(30)^.

La aplicabilidad y eficacia de las puntuaciones pronósticas en pacientes gravemente enfermos con LRA pueden variar debido a las características de esta población, como la presencia de múltiples comorbilidades, inestabilidad hemodinámica y exposición a terapias intensivas. En el presente estudio, abordamos SAPS 3, SOFA y NAS, siendo este último referido a la carga de trabajo del equipo de enfermería. Aunque no mostraron una relación significativa con el tiempo de VMI, evidenciaron una relación significativa con la LRA, como en estudios de cohortes prospectivos brasileños^(22,31)^. Se ha comprobado que el poder discriminatorio del NAS indica una alta demanda de cuidados por parte del equipo de enfermería, y el mayor valor del NAS se ha asociado incluso al desarrollo de LRA en pacientes críticos^(21,22)^.

Nuestro estudio tiene limitaciones, como el hecho de ser una cohorte de un solo centro, y la generalización de nuestros resultados es limitada, con presencia de riesgo de sesgo de medición y tamaño muestral pequeño.

Los hallazgos de este estudio contribuyen significativamente al fortalecimiento de la práctica de enfermería en las UTI, al evidenciar la asociación entre la VMI y la aparición de LRA, especialmente en las etapas más graves de la clasificación KDIGO. Este conocimiento refuerza la importancia de la vigilancia clínica continua realizada por el equipo de enfermería ante alteraciones hemodinámicas y marcadores de laboratorio de la función renal en pacientes críticos con soporte ventilatorio.

Además, los resultados destacan la necesidad de implementar protocolos de enfermería que integren la monitorización temprana de la función renal a los cuidados respiratorios, promoviendo intervenciones oportunas que puedan minimizar la progresión de la LRA. Estas prácticas amplían el alcance de la actuación del enfermero intensivista, favoreciendo las decisiones clínicas basadas en la evidencia, con un impacto directo en la seguridad y los resultados de los pacientes.

## CONCLUSIÓN

El impacto de la ventilación mecánica en pacientes críticos se evidenció por la mayor prevalencia de LRA grave (KDIGO 3) y DRA. El soporte ventilatorio invasivo fue más prevalente entre los ancianos, lo que evidenció la gravedad de los pacientes a partir de SAPS 3 y SOFA y el consiguiente mayor riesgo de muerte. Algunos factores, como los cambios bioquímicos del lactato y la hemoglobina, resultaron significativos entre los pacientes con LRA que recibieron asistencia ventilatoria mecánica invasiva.

El tiempo de ventilación mecánica no se tradujo necesariamente en una mayor carga de trabajo para el equipo de enfermería, ya que, por sí solo, el cuidado de pacientes con LRA repercutió en el aumento del NAS, independientemente del tiempo que permanecieron en ventilación mecánica, lo que destaca que la vigilancia permanente por parte del equipo de enfermería es fundamental para la detección precoz de alteraciones hemodinámicas y de los marcadores de laboratorio de la función renal en pacientes más graves bajo soporte ventilatorio. El desarrollo y la implementación de protocolos son esenciales, ya que permiten intervenciones adecuadas que pueden minimizar la progresión de la LRA, mejorar los resultados clínicos y aumentar la seguridad del paciente.

## Data Availability

Todo el conjunto de datos que apoya los resultados de este estudio fue publicado en el propio artículo.
